# A New Quality Control Strategy for Mori Ramulus Adulteration in Astragali Radix: A Qualitative-Quantitative Coupled Model Based on Non-Targeted Mass Spectrometry Digital Fingerprints and the ElasticNet Algorithm

**DOI:** 10.1021/acsomega.6c04890

**Published:** 2026-08-06

**Authors:** Hao Liang, Hui Wang, Xinjie Shi, Menghan Fan, Minmin Zhou, Bingyan Han, Yun Wang

**Affiliations:** 47839 Beijing University of Chinese Medicine, Beijing 100029, China

## Abstract

Astragali Radix (AR) is widely applied in the pharmaceutical and food fields, but it is highly susceptible to malicious adulteration with the visually highly similar *Mori Ramulus* (MR) during market circulation. To address these issues, This study constructed a qualitative–quantitative coupled adulteration analysis model based on a nontargeted UHPLC-QTOF-MS method by integrating digital fingerprints of AR and MR, ion mapping ratio (IMR) screening, and regression algorithms. Under the experimental conditions in this study, the qualitative screening model established based on IMR could stably detect MR adulteration as low as 5%. Combined with the quantitative prediction model constructed by ElasticNet algorithm (*R* ^2^ = 0.9969, RMSE = 0.0195, MAE = 0.0130), two suspicious adulterated samples were collaboratively screened from 16 batches of AR blind samples (ABS), with predicted MR adulteration ratios of 42.48% and 77.43%, respectively. In conclusion, the digital fingerprints established via nontargeted UHPLC-QTOF-MS coupled with IMR and ElasticNet model can provide a feasible technical support for adulteration screening and the quantitative estimation of the adulteration ratio of AR with MR.

## Graphical abstract

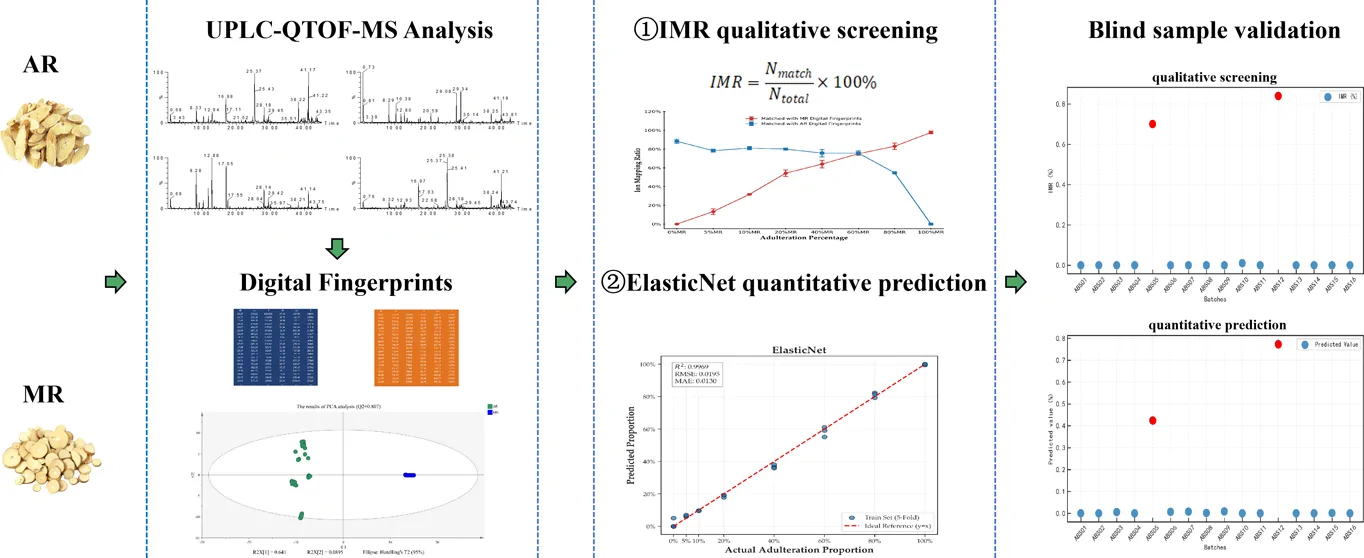

## 1 Introduction

*Astragali Radix* (AR) is the dried root of the leguminous plants *Astragalus membranaceus* (Fisch.) Bge. var. *mongholicus* (Bge.) Hsiao (AMM) and *A. membranaceus* (Fisch.) Bge. (AM) in traditional Chinese medicine (TCM), this herb is frequently utilized as a tonic, esteemed for its dual roles in both medicinal and dietary applications. Traditional therapeutic effects include tonifying qi and elevating yang, consolidating the exterior and stopping sweating, promoting diuresis and reducing swelling, and promoting tissue regeneration and wound healing.^1,2^ AR has been reported to be rich in bioactive constituents, mainly saponins, flavonoids, and polysaccharides, which are associated with multiple pharmacological functions, such as immunomodulatory, anti-inflammatory, antioxidant, and antitumor effects.^3,4^ It is widely used in pharmaceuticals, food, beverages, health supplements and cosmetics.^5,6^

With the continuous expansion in the development and utilization of AR and its related products, the market demand continues to grow.^8,9^ Recently, a novel adulterant of AR, namely *Mori Ramulus* (MR), which is the dried young branch of the Moraceae plant *Morus alba* L., has emerged in the market. Although the two belong to different botanical families and genera and exhibit significant differences in pharmacological effects, MR is inexpensive, and its slice and powder morphologies are highly similar to those of AR, thereby posing a serious threat to the quality stability, clinical efficacy, and medication safety of AR.^10^ Therefore, establishing a rapid, accurate, and highly sensitive identification and detection method for MR adulteration in AR is of great practical significance for regulating the herbal market order and ensuring the safety and efficacy of clinical medication.

Traditional identification methods for AR mainly rely on subjective morphological and microscopic evaluations, which exhibit poor detection capabilities for powder samples and low-proportion adulterated samples, making it difficult to cope with increasingly concealed adulteration practices.^11–13^ To this end, researchers have established various modern identification and quality evaluation methods for the quality control of AR, which mainly encompass DNA molecular authentication, spectral and imaging technologies, and chromatographic analysis methods. Among DNA molecular authentication technologies, DNA barcoding, represented by ITS and matK gene sequencing, is less affected by factors such as geographical origin, harvest period, and growth environment, enabling precise identification of herbal materials at the botanical origin and species levels.^14,15^ Spectral and imaging technologies, such as Fourier transform infrared (FT-IR) spectroscopy, diffuse reflectance infrared Fourier transform spectroscopy (DRIFTS), surface-enhanced Raman spectroscopy (SERS), and hyperspectral imaging (HSI), are suitable for the rapid and nondestructive preliminary screening of AR samples, demonstrating promising application prospects in the geographical origin traceability and adulteration screening of AR;^16–19^ additionally, conventional chromatographic techniques are widely applied in the quality evaluation research of AR.^20,21^ However, all of these existing technologies exhibit application limitations, making it difficult to simultaneously address the three requirements of trace adulteration screening, quantitative estimation of adulteration proportions, and anti-interference in complex matrices. Therefore, there is an urgent need to establish an analytical strategy that integrates precise qualitative identification and quantitative resolution capabilities for the accurate detection of MR adulteration in AR materials.

In recent years, liquid chromatography–mass spectrometry (LC–MS) technologies, represented by UHPLC-QTOF-MS, have become pivotal technologies for component analysis, fingerprint construction, and quality control in complex traditional Chinese medicine systems owing to their high separation efficiency, high mass accuracy, and high sensitivity.^22,23^ The digital identity strategy based on LC–MS data has been successfully applied to the precise identification and traceability of medicinal materials such as ginseng, American ginseng, and tangerine peel.^24–26^ To address the lack of a dedicated analytical workflow for detecting MR adulteration in AR powders, we hypothesized that multibatch, mutually exclusive UHPLC-QTOF-MS fingerprints of AR and MR would support both qualitative screening and quantitative estimation under a fixed analytical workflow.^7^ We therefore (i) established AR- and MR-specific digital fingerprints from multibatch samples, (ii) developed an ion mapping ratio (IMR) based operational rule for qualitative screening, and (iii) built and compared regression models for estimating MR proportion in samples.

## 2 Materials and Methods

### 2.1 Herbal Materials

A total of 40 batches of AR were collected from Gansu Province, Shanxi Province, the Inner Mongolia Autonomous Region and Jilin Province in China: 20 of AMM and 20 of AM. Twenty-five batches of MR were collected from the provinces of Sichuan, Jiangsu and Zhejiang in China. Researcher Wang Yun identified all 65 batches of medicinal materials and found them to comply with the standards specified in the Pharmacopoeia of the People’s Republic of China.^27^ For this study, 24 positive adulterated samples were prepared independently with varying adulteration ratios, and 16 batches of AR blind samples (ABS) were purchased from markets. All samples material information is detailed in Table S1.

### 2.2 Experimental Consumables

LC–MS grade acetonitrile (Lot: R142988) and methanol (Lot: EK243-US) were purchased from Honeywell Trading (Shanghai) Co., Ltd. (Shanghai, China). LC–MS grade formic acid (Lot: 8387760) was purchased from DIKMA Technologies Inc. (Foothill Ranch, CA, USA). Purified water was prepared in-house with a Milli-Q ultrapure water purification system (Merck KGaA, Darmstadt, Germany). Sterile syringes (2 mL, Lot: 240401) were obtained from Jumin Biotechnology Co., Ltd. (Shanghai, China), whereas PTFE syringe filters (13 mm, 0.22 μm, Lot No. F242903) were obtained from DIKMA Technologies Inc. (Foothill Ranch, CA, USA) and MS-grade glass sample vials were obtained from Shimadzu (China) Co., Ltd. (Shanghai, China) and Waters Corporation (Milford, MA, USA), respectively.

### 2.3 Chromatographic and Mass Spectrometry Conditions

UHPLC-QTOF-MS analyzes were carried out on an ACQUITY UHPLC system coupled with a Xevo G2-XS QTOF mass spectrometer (Waters Corporation, Milford, MA, USA). Chromatographic separation was achieved on an Acquity UPLC HSS T3 column (2.1 mm × 100 mm, 1.7 μm). The column oven was maintained at 35 °C, whereas the autosampler temperature was set at 25 °C. A 2 μL aliquot was injected for each run, and the flow rate was fixed at 0.3 mL/min. Mobile phase A consisted of methanol containing 0.1% formic acid, while mobile phase B was water with 0.05% formic acid. The elution program started at 5% A and was held for 0.5 min, then increased to 15% at 5.5 min, to 50% at 23.5 min, and further to 95% at 40.5 min. This composition was maintained until 42.0 min, after which it was returned to 5% A at 42.1 min and equilibrated to 45.0 min. Mass spectral data were collected in MSE mode using an electrospray ionization source operated in both positive and negative ionization modes. The acquisition range was *m*/*z* 50–1500 with a scan time of 0.1 s. The capillary voltage was set to 3.0 kV in positive mode and 2.5 kV in negative mode. The remaining MS parameters were as follows: cone voltage, 40 V; source offset, 80 V; source temperature, 120 °C; desolvation temperature, 300 °C; desolvation gas flow, 600 L/h; and collision energy, 10–50 eV.

### 2.4 Sample Pretreatment

Take samples of AM, AMM, and MR from each batch. Cut them into decoction pieces and grind them using a high-speed pulverizer. Sieve the mixture through a No. 3 sieve (pore size: 355 μm ± 13 μm). Weigh out equal amounts of powder from each batch, then mix them thoroughly to prepare AR and MR mixed samples. The MR mixed samples were then adulterated into the AR mixed sample at different mass ratios in order to prepare positive adulterated samples with MR mass fractions of 0%, 5%, 10%, 20%, 40%, 60%, 80% and 100%. Three parallel preparations were made for each adulteration ratio. Commercially available decoction pieces of AR were pulverized and sieved using the same method for later use. Precisely weigh 0.5 g of each of the pure powder samples, the positive adulterated samples and the blind samples. Place each sample in a stoppered conical flask and accurately add 25 mL of a 70% methanol aqueous solution. Seal the flasks tightly and perform ultrasonic extraction for 20 min (power: 500 W; frequency: 40 kHz). After extraction, remove the flask and allow it to cool to room temperature (25 °C). The supernatant was filtered through a 0.22 μm microporous membrane, and the collected filtrate was utilized as the test sample.

### 2.5 Digital Fingerprints Construction and IMR-Based Qualitative Screening

This method uses UHPLC-QTOF-MS detection data to create digital fingerprints for AR and MR. Qualitative screening of MR adulteration in AR is achieved through IMR calculations. The algorithm converts the chemical characteristics of different herbal materials into a three-dimensional digital ion matrix comprising retention time (*t*_R_), mass-to-charge ratio (*m*/*z*), and ion intensity (*I*). The overall process comprises five core stages: data preprocessing, common ion matrix construction, specific ion matrix construction,digital fingerprints generation, and adulteration qualitative screening (shown in Scheme 1). Throughout the workflow, data conversion utilizes Waters Progenesis QI analysis software (version 2.4.69). The analysis conditions use high-resolution mode with unified settings: ESI + ionization mode; retention time range of 1.00–42.00 min; and an automatic peak detection threshold.

**Scheme 1:**
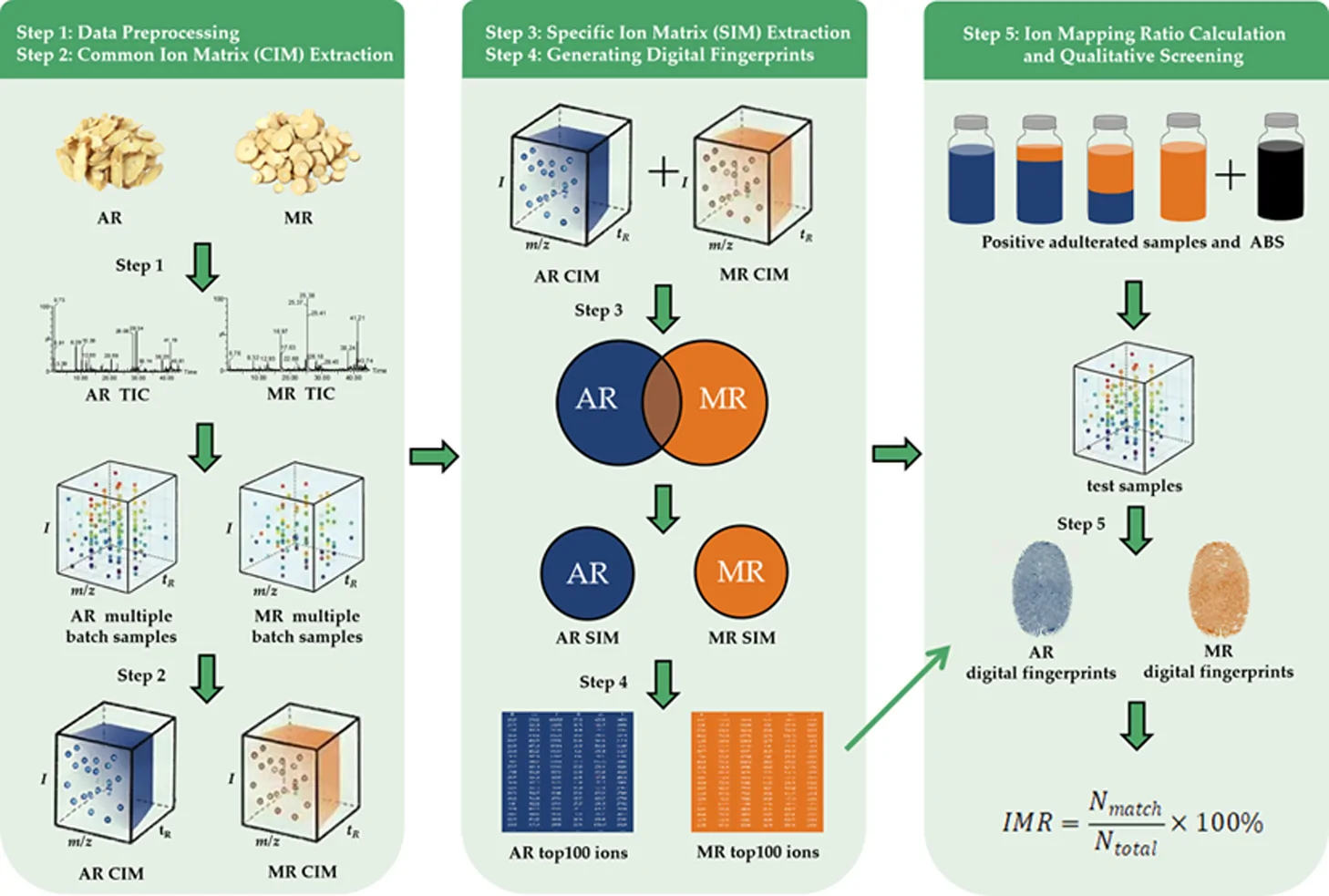
Digital Fingerprints Identification Pathway Diagram

#### 2.5.1 Step 1: Data Preprocessing

Raw ion data from UHPLC-QTOF-MS analyses of AMM, AM, MR, positive adulteration sample from different batches and blank extraction solvents (70% methanol aqueous solutions) were converted into digital matrices (DM) with dimensions of *n* × 3 (where *n* is the number of ions and the columns represent *t*_R_, *m*/*z*, and *I*, respectively). Formula

$$DM={\left[\begin{array}{lll} t_{R,1} & {\left(m/z\right)}_{1} & I_{1}\\ t_{R,2} & {\left(m/z\right)}_{2} & I_{2}\\ \vdots & \vdots & \vdots \\ t_{R,n} & {\left(m/z\right)}_{n} & I_{n} \end{array}\right]}_{n\times 3}$$

This study regarded background ions from the 70% methanol aqueous solution present in all samples as interference. Using exclusion criteria of Δ*t*_R_ ≤ 0.10 min and Δ*m*/*z* ≤ 0.01, ions in the herbal samples that shared a similar *t*_*R*_ and *m*/*z* with the blank extraction solvent were removed. This generated a background-corrected ion matrix.

#### 2.5.2 Step 2: Common Ion Matrix (CIM) Extraction

Common ions represent stable, shared information across different batches of herbal materials. They serve as core indicators that characterize their fundamental chemical features. For multiple batches of AMM and AM, this study set matching thresholds of Δ*t*_*R*_ ≤ 0.10 min and Δ*m*/*z* ≤ 0.01. Based on python (version 3.12.7), intersection operations were performed on their background-corrected ion matrices to extract the CIM to AR. Similarly, the same logic and parameters were applied to construct CIM for multiple batches of MR.

#### 2.5.3 Step 3: Specific Ion Matrix (SIM) Extraction

Specific ions are defined as components that are unique to the target herbal material and absent in the reference material. These ions serve as the basis for identifying adulteration. First, based on Python (version 3.12.7), an intersection operation is performed on the AR CIM and MR CIM to obtain the common ion matrix of AR and MR.Subsequently, using a set difference strategy, the common ion matrix of AR and MR is subtracted from the AR CIM to obtain the AR SIM. Similarly, the common ion matrix of AR and MR is subtracted from the MR CIM to obtain the MR SIM.

#### 2.5.4 Step 4: Generating Digital Fingerprints

From the SIM of AR and MR, the top 100 [*t*_*R*_-*m/z*-*I*] ranked by response intensity were selected to construct the final digital fingerprints representing the digital information of the herbal materials.

#### 2.5.5 Step 5: IMR Calculation and Qualitative Screening

IMR calculation: After the test sample undergoes identical pretreatment and UHPLC-QTOF-MS detection, the background-subtracted ion matrix is obtained using the aforementioned method. Matching is performed on an ion-by-ion basis against the digital fingerprints of the AR and MR samples using the following criteria: Δ*t*_*R*_ ≤ 0.10 min and Δ*m*/*z* ≤ 0.01. The IMR is then calculated to evaluate sample matching with the AR and MR digital fingerprints. IMR represents the proportion of successfully matched ions between the test sample and the matrix of digital fingerprints and is calculated as follows

$$IMR=\frac{N_{match}}{N_{total}}\times 100\%$$

where IMR is the ion mapping ratio; *N*_match_ represents the number of successfully matched characteristic ions between the test sample and the digital fingerprints; and *N*_total_ is the total number of characteristic ions contained in the digital fingerprints.

Qualitative screening: If the IMR between the test sample and the MR digital fingerprints matrix exceeds the decision threshold, the sample is considered adulterated with MR. A higher IMR with the AR digital fingerprints indicates greater purity of the genuine sample.

### 2.6 Quantitative Prediction Based on Regression Models

#### 2.6.1 Data Preparation

First, three samples each of AM, AMM, and MR were randomly selected and combined with all positive samples with adulteration proportions ranging from 5% to 80% to constitute the training set. Subsequently, taking Δ*t*_*R*_ ≤ 0.10 min and Δ*m*/*z* ≤ 0.01 as the criterion, the feature ions in the digital fingerprints of AR and MR were integrated to construct a feature ion set. Based on this feature ion set, the *I* values of the corresponding ions in each sample were extracted to establish a feature matrix. A half-minimum imputation strategy was applied to the missing or zero values within the feature matrix.

#### 2.6.2 Model Construction

To quantitatively predict the adulteration proportion of MR in AR, this study employed OPLS-R, SVR, RFR, and ElasticNet to construct prediction models. Among them, OPLS-R makes predictions by separating latent variables that are correlated with the dependent variable from those that are orthogonal (uncorrelated) to it; SVR utilizes kernel functions to fit complex nonlinear relationships, thereby achieving prediction; RFR performs modeling and prediction by ensembling multiple decision trees; and ElasticNet combines L_1_(Lasso) and L_2_(Ridge) regularization to perform prediction by achieving variable selection and parameter shrinkage.^28–31^ All models employed 5-fold cross-validation (random seed = 42) for hyperparameter tuning to objectively evaluate their predictive performance.^32^

#### 2.6.3 Model Evaluation

In this study, the predictive performance of four regression models was evaluated using three metrics: the coefficient of determination (*R* ^2^), root-mean-square error (RMSE), and mean absolute error (MAE). Among these, *R* ^2^ reflects the model’s explanatory capacity for the dependent variable, with a value closer to 1 indicating a better goodness-of-fit; RMSE and MAE quantify the overall deviation and absolute error between the predicted and actual values, respectively, where smaller values for both metrics denote higher predictive accuracy of the model.^33,34^ Based on these evaluation results, the algorithm with the optimal performance was selected to construct the final quantitative prediction model, which was subsequently applied to 16 batches of ABS for exploratory application analysis. All the aforementioned data modeling and evaluation procedures were implemented using Python (version 3.12.7).

## 3 Results

### 3.1 Results of UHPLC-QTOF-MS Analysis

In this study, optimized UHPLC-QTOF-MS experimental conditions were employed to collect data on multiple batches of AR, MR, and various gradient-spiked positive samples in the positive ion (ESI+) mode. Figure 1 shows the mass spectra of representative samples obtained under the same sample pretreatment and experimental conditions, including blank solvent, AM, AMM, MR, and a positive adulterated sample (60% MR). As shown in Figure 1, the spectra exhibit high peak resolution, stable baselines, and good response for low-abundance ions, comprehensively and objectively characterizing the complex chemical composition of each sample. A direct comparison of the AR and MR spectra reveals inherent differences in the distribution of peak retention times and the relative abundances of major ion peaks, reflecting their respective characteristic chemical profiles. However, due to the complexity of the TCM matrix, coupled with natural fluctuations in plant secondary metabolites caused by environmental factors such as origin and harvest season, relying solely on visual comparison of raw mass spectra or simply on targeted monitoring of a few known peaks is highly prone to misinterpretation.

**Figure 1:**
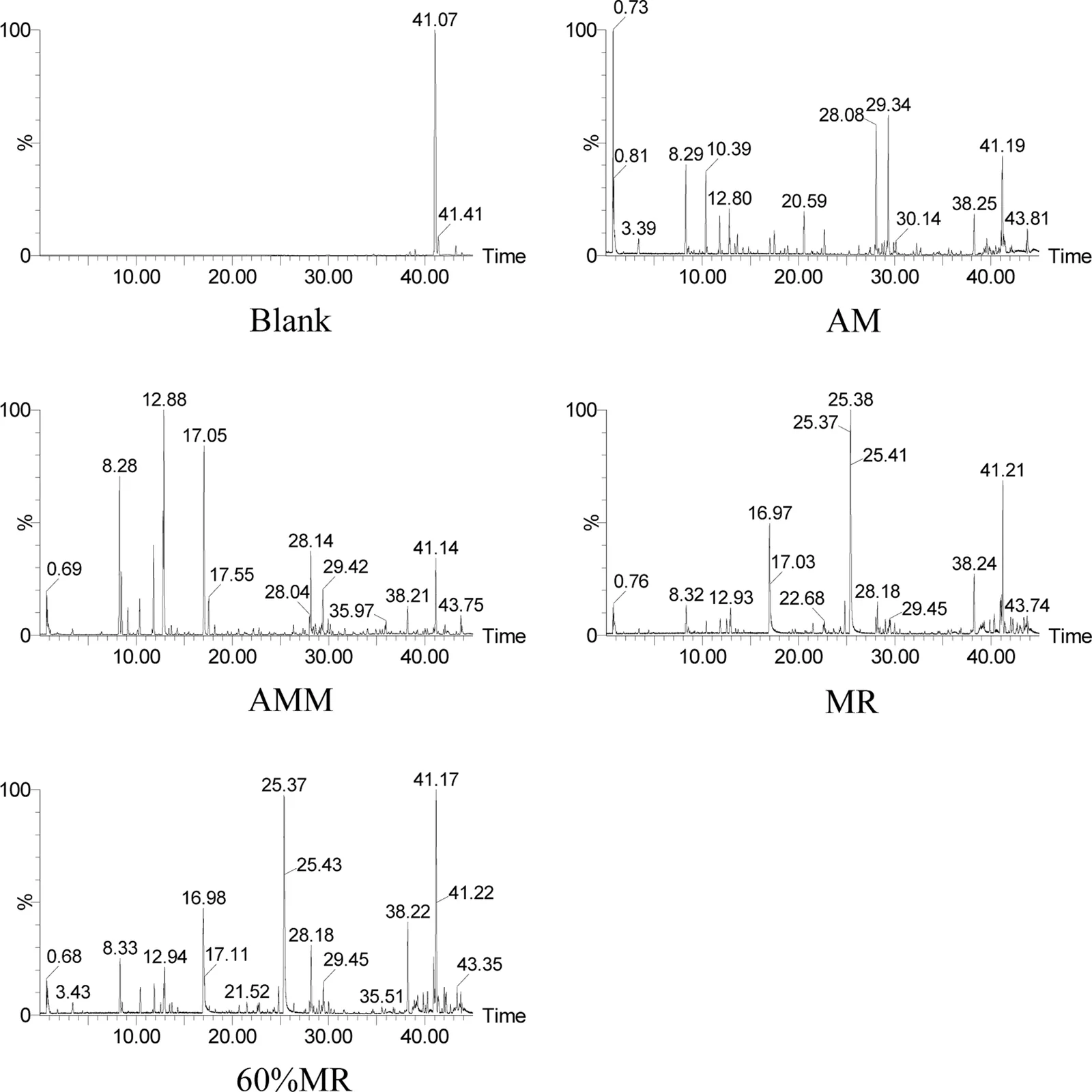
Base-peak chromatograms of blank, AMM, AM, MR and positive adulterated sample (60% MR).

Furthermore, as observed in the spectrum of the positive adulterated sample (60% MR), despite the high proportion of MR (60%), the overall mass spectrum of this positive spiked sample still exhibits a high degree of visual similarity to that of the AM and AMM, making it impossible to quantitatively assess the extent of adulteration. Therefore, to fully extract specific differential information from the mass spectra, it is imperative to digitize the aforementioned mass spectra.

### 3.2 Results of Digital Processing

The raw mass spectrometry data from the 70% methanol–water blank solution and each herbal sample underwent peak alignment and extraction processing using Progenesis QI software. The background subtraction process was executed on the herbal samples, with a total of 1428 [*t*_*R*_-*m/z-I*] derived from the blank solution. Subsequent to processing, ion matrices were obtained for 20 batches of AMM, 20 batches of AM, and 25 batches of MR, with the removal of background signals. To construct a standard digital fingerprint, the data set was randomly partitioned proportionally: To establish the digital fingerprints, 10 batches each of AMM, AM, and MR were selected at random, while the remaining 10 batches of AMM, 10 batches of AM, and 15 batches of MR were allocated to the identification verification set. The ion matrix results of the samples used for constructing digital fingerprints showed that there were significant inter- and intragroup variations in the number of [*t*_*R*_-*m/z-I*] feature ions across different batches (see Tables 1 and 2). As a case in point, the feature ion count of AM fluctuated dramatically between batches (e.g., 4107 for AM02 vs 5822 for AM17). By contrast, AMM and MR exhibited smaller interbatch variations (e.g., 6559 for AMM07 and 7721 for AMM19; 3857 for MR02 and 4040 for MR08). This variation is an objective reflection of the inherent chemical composition of the herbal materials. The substantial disparities in AM feature ions imply that its chemical composition is more susceptible to environmental factors, including its provenance, storage conditions, and individual development. Therefore, to avoid matrix bias from single or limited batches, constructing universally applicable digital fingerprints must incorporate sample information from multiple batches and origins.

**Table 1:** Number of [*t*_*R*_-*m*/*z*-*I*] Feature Ions in AR Samples

name	batch	number	name	batch	number
AM	AM02	4107	AMM	AMM03	6914
	AM06	4827		AMM05	7021
	AM08	4968		AMM07	6559
	AM10	4936		AMM10	6777
	AM13	5514		AMM13	6734
	AM15	5810		AMM15	6737
	AM16	5736		AMM16	6798
	AM17	5822		AMM17	6876
	AM18	5671		AMM19	7721
	AM20	5777		AMM20	7359

**Table 2:** Number of [*t*_*R*_-*m*/*z*-*I*] Feature Ions in MR Samples

name	batch	number
MR	MR02	3857
	MR05	4121
	MR08	4040
	MR10	4030
	MR13	3978
	MR15	4018
	MR17	3950
	MR21	3856
	MR24	4020
	MR25	3614

### 3.3 Construction of Digital Fingerprints

Based on the benchmark training set and a predetermined screening strategy, the top 100 core [*t*_*R*_-*m/z*-*I*] features with the highest response intensities were extracted to construct standard digital fingerprints for AR and MR (Tables S2 and S3). Subsequently, the IMR formula was applied to calculate and evaluate the match between samples in the identification verification set and the standard digital fingerprints. The specific results are shown in Tables 3 and 4.The results demonstrated that the IMR distribution for the 20 AR validation samples matched against the AR digital fingerprints fell within a high-fit range of 70%–91%, while the cross-match IMR against the MR digital fingerprints was nearly entirely 0% (with only one instance at 1%). In a similar vein, the IMR distribution for the 15 MR validation samples matched against their MR digital fingerprints ranged from 93% to 101%, while the IMR for matching against the AR digital fingerprints was predominantly 0% (with only one instance at 1%). The results showed that the IMR of AR and MR validation samples were relatively high when matched with their respective exclusive digital fingerprints, while the cross-matching IMR approached zero.

**Table 3:** IMR of AR Validation Samples Matched with AR and MR Digital Fingerprints

name	batches	IMR of matched with AR digital Fingerprints\1	IMR of matched with MR digital Fingerprints\1
AM	AM01	82%	0%
	AM03	71%	0%
	AM04	87%	0%
	AM05	91%	0%
	AM07	90%	0%
	AM09	86%	0%
	AM11	71%	0%
	AM12	73%	1%
	AM14	74%	0%
	AM19	70%	0%
AMM	AMM01	79%	0%
	AMM02	80%	0%
	AMM04	81%	0%
	AMM06	78%	0%
	AMM08	87%	0%
	AMM09	79%	0%
	AMM11	85%	0%
	AMM12	86%	0%
	AMM14	90%	0%
	AMM18	74%	0%

**Table 4:** IMR of MR Validation Samples Matched with AR and MR Digital Fingerprints

name	batches	IMR of matched with AR digital Fingerprints\1	IMR of matched with MR digital Fingerprints\1
MR	MR01	1%	93%
	MR03	0%	101%
	MR04	0%	96%
	MR06	0%	99%
	MR07	0%	98%
	MR09	0%	100%
	MR11	0%	100%
	MR12	0%	99%
	MR14	0%	95%
	MR16	0%	99%
	MR18	0%	99%
	MR19	0%	97%
	MR20	0%	97%
	MR22	0%	98%
	MR23	0%	98%

It is worth noting that the IMR of certain samples (such as MR03) exhibited values exceeding 100%. To investigate the underlying causes, we conducted a similarity comparison of the characteristic ions in the digital fingerprints of AR) and MR, respectively, within the predefined mass tolerance window. The results showed that within this tolerance range, there was no overlapping matching phenomenon among the characteristic ions within the two groups (100 ions each), thereby ruling out the interference caused by excessively high similarity within the digital fingerprints. Further analysis indicated that under the established tolerance range, a single characteristic ion in the digital fingerprints could repeatedly match multiple ion peaks in the sample, which is the direct cause of the IMR value reaching 101%. Taking the MR03 sample as an example, there were two adjacent ion peaks in its chromatogram (*t*_R_ = 9.02 min, *m*/*z* = 492.3164 and *t*_R_ = 8.94 min, *m*/*z* = 492.3168), both of which matched the same characteristic ion in the MR digital fingerprints (*t*_R_ = 9.00 min, *m*/*z* = 492.32). This multiple matching phenomenon only causes minor deviations in the calculation results of a very limited number of samples, and will not have a substantial impact on the final conclusion of adulteration identification.

To evaluate whether these digital fingerprints reflect the chemical differences between AR and MR, principal component analysis (PCA) was performed using SIMCA 14.1. The *t*_R_-*m*/*z* pairs were defined as ion-feature variables, and their corresponding intensities (*I*) were used as feature values to construct the data matrix. As shown in the PCA score plot (Figure 2), samples from the 40 AR and 25 MR batches clearly separated into two nonoverlapping clusters, indicating that these two herbal materials possess distinct digital fingerprints. The PCA model exhibited robust explanatory and predictive capabilities (*R* ^2^ *X* = 0.840 and *Q* ^2^ = 0.807). Thus, digital fingerprints can effectively distinguish between AR and MR, two easily confused herbal materials. This result further validates the reliability of digital fingerprints in differentiating the two herbal materials. Even if the IMR exhibits minor fluctuations (e.g., reaching 101% in batch MR03), digital fingerprints will not cause misidentification due to individual sample variations. The results from Tables 3 and 4 and Figure 2 confirm that the digital fingerprints established in this study possess both intraspecific commonalities and interspecific specificity.

**Figure 2:**
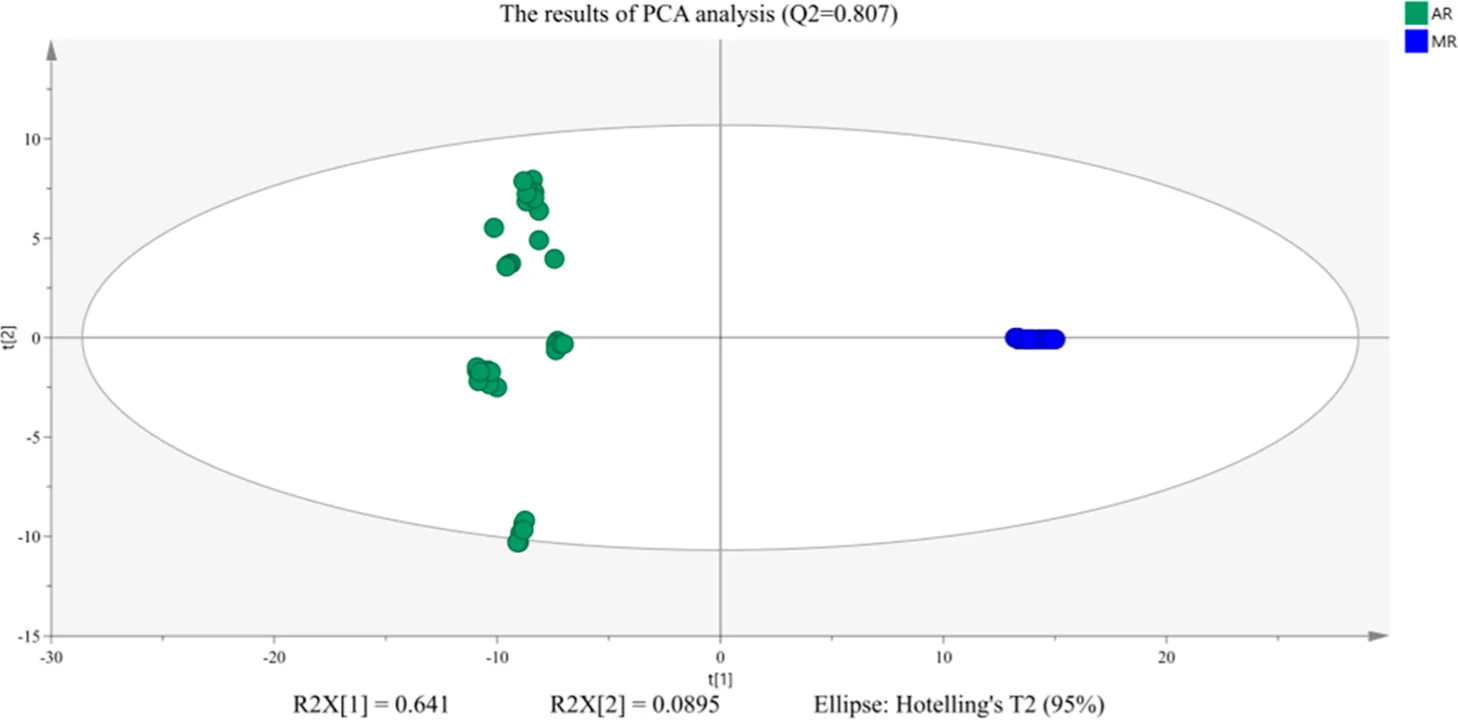
PCA score plot of AR and MR samples.

### 3.4 Results of Qualitative Screening Model

After constructing the digital fingerprints of AR and MR, matching was performed on the gradient positive adulterated samples, with the results detailed in Tables 5 and 6. As shown in Figure 3, when the positive adulterated samples were compared with the AR digital fingerprints, the IMR overall decreasing trend with local fluctuations and a downward trend with the elevation of the MR proportion; specifically, the IMR of the 0% MR group was no less than 87%, whereas that of the 100% MR group dropped to 0%. Conversely, when the gradient adulterated samples were matched against the MR digital fingerprints, the IMR exhibited a significant positive correlation trend with the increase in the MR adulteration proportion. The IMR of the 0% MR group was 0%, and that of the 100% MR group was no less than 96%.

**Table 5:** IMR of Positive Adulterated Samples Matched with AR Digital Fingerprints (*n* = 3)

sample	IMR-1	IMR-2	IMR-3	average IMR	SD
0% MR	91%	87%	87%	88.3%	2.31%
5% MR	77%	80%	78%	78.3%	1.53%
10% MR	82%	82%	79%	81.0%	1.73%
20% MR	81%	80%	79%	80.0%	1.00%
40%MR	80%	75%	72%	75.7%	4.04%
60% MR	73%	77%	77%	75.7%	2.31%
80% MR	55%	55%	54%	54.7%	0.58%
100% MR	0%	0%	0%	0.00%	0.00%

**Table 6:** IMR of Positive Adulterated Samples Matching with MR Digital Fingerprints (*n* = 3)

sample	IMR-1	IMR-2	IMR-3	average IMR	SD
0% MR	0%	0%	0%	0.0%	0.00%
5% MR	14%	10%	16%	13.3%	3.06%
10% MR	32%	32%	31%	31.7%	0.58%
20% MR	54%	51%	58%	54.3%	3.51%
40% MR	60%	64%	68%	64.0%	4.00%
60% MR	77%	74%	74%	75.0%	1.73%
80% MR	85%	79%	85%	83.0%	3.46%
100% MR	99%	98%	96%	97.70%	1.53%

**Figure 3:**
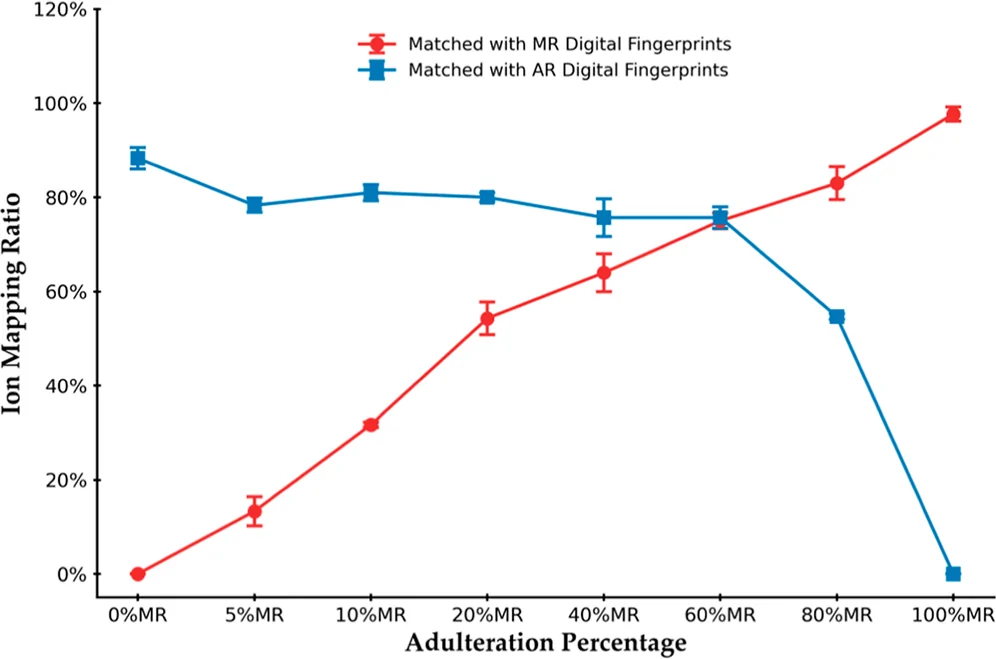
Trend of IMR for positive adulterated samples (0%–100% MR) matched with the digital fingerprints of MR and AR, respectively. (Error bars denote the standard deviation (SD) of three independent measurements).

To clarify the rationale for establishing the decision threshold for screening MR adulteration, this study analyzed the distribution characteristics of the MR-IMR. The MR-IMR values for the AR validation samples and the 0% MR adulterated samples ranged from 0% to 1%, whereas the MR-IMR values for the 5% MR adulterated samples were distributed within the range of 10%–16%. The value of 10.0% represented the lowest MR-IMR detected in the 5% MR adulterated samples, which was significantly higher than the upper limit of the unadulterated samples (1%). Furthermore, utilizing this minimum value as a benchmark maximizes the detection rate for low-proportion adulterated samples, thereby effectively circumventing the risks of false negatives and false positives; consequently, MR-IMR ≥10.0% was preliminarily established as the decision threshold for MR adulteration.

Subsequently, receiver operating characteristic (ROC) curve analysis was performed using MR-IMR as the categorical variable. The AR validation samples and 0% MR adulterated samples were assigned to the negative group (*n* = 23), while the 5%–100% gradient adulterated samples were assigned to the positive group (*n* = 21). The results showed (Figure S1) that the area under the ROC curve (AUC) was 1.000, indicating that this indicator can completely distinguish between adulterated and unadulterated samples. When the threshold was set at MR-IMR ≥10.0%, all positive and negative samples were correctly discriminated. Both the sensitivity and specificity of the method reached 100.0% (95% CI: 83.9%–100.0% and 85.2%–100.0%, respectively). Therefore, MR-IMR ≥10.0% can serve as a reliable decision threshold for screening MR adulteration in AR under the conditions of this study.

In this study, blind testing was conducted on 16 batches of ABS using digital fingerprints, with the detailed results presented in Table 7. The analytical results demonstrated that the IMR of samples ABS05 and ABS12, when matched against the MR digital fingerprints, were 70.0% and 84.0%, respectively, which far exceeded the 10.0% detection threshold. This result indicates a adulteration in the aforementioned commercial blind samples, suggesting the presence of MR as an exogenous substance. For the remaining 14 batches of samples, the IMR values matched against the MR digital fingerprints were all below the detection threshold (IMR ≤1.0%), and their IMR values matched against the AR digital fingerprints were all ≥70.0%; consequently, no MR adulteration was observed in these samples.

**Table 7:** IMR of ABS Matched with AR and MR Digital Fingerprints

name	batches	IMR of matched with AR digital Fingerprints\1	IMR of matched with MR digital Fingerprints\1
blind samples	ABS01	84%	0%
	ABS02	89%	0%
	ABS03	92%	0%
	ABS04	92%	0%
	ABS05	88%	70%
	ABS06	85%	0%
	ABS07	70%	0%
	ABS08	89%	0%
	ABS09	84%	0%
	ABS10	86%	1%
	ABS11	83%	0%
	ABS12	51%	84%
	ABS13	92%	0%
	ABS14	84%	0%
	ABS15	91%	0%
	ABS16	75%	0%

### 3.5 Results of Quantitative Prediction Model

After data preprocessing, a mass spectrometry feature matrix with dimensions of 27 × 200 was constructed as the training set. To determine the optimal algorithm for the quantitative analysis of the adulteration proportion of MR in AR, this study rigorously evaluated four regression models (OPLS-R, SVR, RFR, and ElasticNet) on the training set using 5-fold cross-validation combined with a hyperparameter tuning strategy. The performance metrics (R2, RMSE, MAE) and optimal hyperparameters of each model are detailed in Table 8. The detailed hyperparameter search spaces, along with the software packages and corresponding versions utilized in the analysis, are provided in Tables S5 and S6. The *R* ^2^ values of the four models ranged from 0.95 to 0.99, all demonstrating good quantitative prediction capabilities. Among them, the ElasticNet model effectively overcame the challenges of high dimensionality and multicollinearity associated with mass spectrometry variables, achieving the highest R2 (0.9969) along with the lowest RMSE (0.0195) and MAE (0.0130), thereby exhibiting the optimal predictive performance (see the regression scatter plot in Figure 4a). Therefore, this study ultimately selected ElasticNet to construct the quantitative regression model. Driven by the regularization properties of ElasticNet, 52 feature ions (detailed in Table S4) that contributed significantly to the adulteration analysis were selected from the feature matrix based on their regression coefficient values (RCV). A larger absolute value of the regression coefficient (|RCV|) indicates a greater contribution of the feature ion to the prediction of the dependent variable, allowing it to serve as a candidate marker for adulteration identification. Figure 4b displays the top 10 important feature ions ranked by the |RCV|. Among them, feature ions with positive RCV were attributed to MR, such as F101 (*m*/*z* 423.1806, *t*_*R*_ 24.74 min, RCV = 0.0269), F120 (*m*/*z* 679.2556, *t*_*R*_ 29.36 min, RCV = 0.0243), and F023 (*m*/*z* 595.3496, *t*_*R*_ 12.47 min, RCV = 0.0218), whose *I* values increased progressively with the elevation of the adulteration proportion. Conversely, feature ions with negative RCV were attributed to AR, such as F064 (*m*/*z* 943.5319, *t*_*R*_ 20.65 min, RCV = −0.0211) and F025 (*m*/*z* 463.1598, *t*_*R*_ 12.79 min, RCV = −0.0180), whose *I* values were gradually diluted and decreased as the degree of adulteration increased.

**Table 8:** Performance Metrics and Optimal Hyperparameters of Model

model	R2	RMSE	MAE	hyperparameter
OPLS-R	0.9622	0.0683	0.0266	opls_n_components: 1
				pls_n_components: 1
SVR	0.9517	0.0771	0.0635	svr_kernel: linear
				svr_C: 0.1
RFR	0.9845	0.0437	0.0351	rf__max_depth: None
				rf__n_estimators: 100
ElasticNet	0.9969	0.0195	0.0130	alpha: 0.004316
				l1_ratio: 0.1

**Figure 4:**
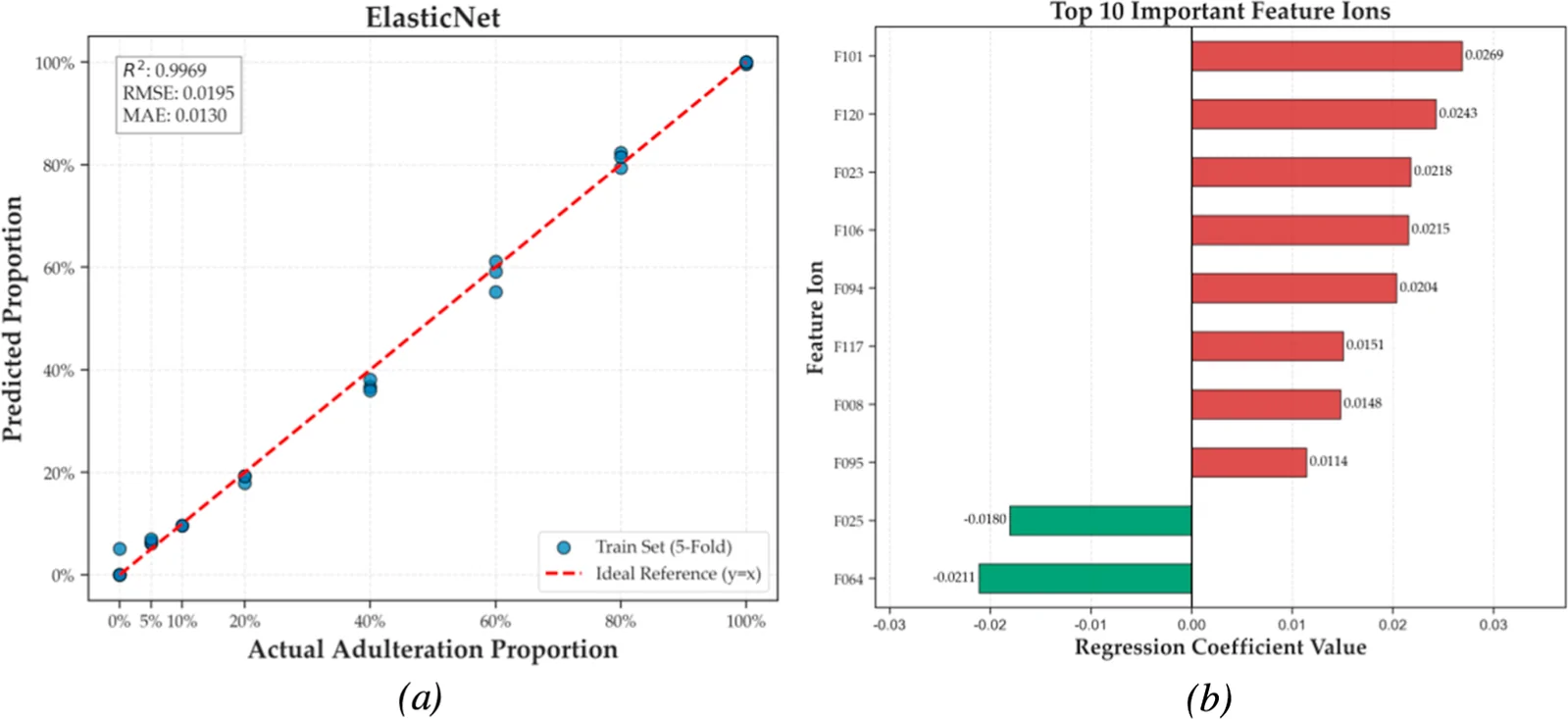
Quantitative prediction of MR adulteration in AR using the ElasticNet model. (a) Regression scatter plot of actual versus predicted adulteration proportions. Blue dots represent the training set (5-fold cross-validated) and the red dashed line indicates the ideal reference line (*y* = *x*). (b) Weight plot of the top 10 important feature ions ranked by the absolute values of their regression coefficients. Red positive bars represent feature ions originating from MR, while green negative bars represent feature ions originating from AR. Numerical values at the end of each bar indicate the RCV.

To evaluate the generalization capability of the model, 16 batches of ABS were further input into the ElasticNet model for exploratory application analysis, with the prediction results presented in Table 9. The results indicated that samples ABS05 and ABS12 were quantitatively predicted to contain 42.48% and 77.43% MR adulteration, respectively, whereas the predicted values for the remaining ABS batches approached zero, and these quantitative prediction results further suggesting the presence of MR adulteration in these two ABS samples.

**Table 9:** Quantitative Prediction of MR Adulteration in ABS Using the ElasticNet Model

samples	predicted value	samples	predicted value
ABS01	0.00%	ABS09	0.88%
ABS02	0.00%	ABS10	0.00%
ABS03	0.57%	ABS11	0.00%
ABS04	0.00%	ABS12	77.43%
ABS05	42.48%	ABS13	0.00%
ABS06	0.67%	ABS14	0.00%
ABS07	0.79%	ABS15	0.13%
ABS08	0.17%	ABS16	0.00%

## 4 Discussion

### 4.1 Discussion of UHPLC-QTOF-MS Experimental Conditions

UHPLC-QTOF-MS has become a key data acquisition platform for the comprehensive chemical profiling and digital authentication of TCM because of its excellent separation capability, high sensitivity, and accurate mass measurement.^35–37^ This technique enables the rapid separation and characterization of hundreds of constituents in highly complex herbal matrices, while also allowing the detection of low-abundance characteristic ions. In the present study, both sample pretreatment and instrumental conditions were evaluated during method development. For sample preparation, AR and MR were pulverized and passed through a No. 3 sieve before the preparation of adulterated positive samples. This procedure not only ensured accurate control of the adulteration ratios, but also better reflected practical testing scenarios for commercially available products such as AR powder and formula granules.

With respect to extraction conditions, methanol, 70% methanol, 50% methanol, ethanol, and water were individually evaluated for their ability to extract the chemical constituents of AR and MR. Among the tested solvents, 70% methanol produced stronger mass spectrometric responses and provided richer chemical information, showing a clear advantage over the other extraction media. Two extraction approaches, namely ultrasonic extraction and heat-reflux extraction, were then compared. No significant difference in extraction efficiency was observed between these two methods; however, ultrasonic extraction was more convenient to perform and offered higher processing efficiency together with better batch-to-batch stability. Accordingly, 70% methanol combined with 20 min of ultrasonic extraction was selected as the final sample pretreatment procedure.

In the context of mass spectrometry acquisition optimization, this study comparatively analyzed the ESI+ and ESI– ionization modes. The findings suggested that the ESI + mode resulted in a greater number of ion species and more stable peak responses. Consequently, this method is particularly well-suited for the chemical composition analysis of AR and MR samples. When employed in conjunction with the MSE data nondependent sampling mode, the acquisition of parent and fragment ion information can be achieved in a simultaneous manner during a single injection. This approach was found to be effective in preserving the full range of mass spectrometry characteristics of the sample. Consequently, it provides a more comprehensive data foundation for subsequent digital processing and fingerprints construction. Moreover, the present study examined the impact of three collision energy conditions (10–30 eV, 10–40 eV, and 10–50 eV) on fragment information acquisition. The findings suggest that the 10–50 eV condition generates the most comprehensive fragment ion information and effectively reflects the chemical composition characteristics of AR and MR. Consequently, this voltage range was identified as the optimal collision energy for the present study.

In summary, the optimization of these experimental conditions establishes a reliable foundation for subsequent digital fingerprint construction and adulterant identification.

### 4.2 Discussion of Digital Fingerprints

This study successfully generated digital fingerprints for AR and MR using nontargeted mass spectrometry data obtained via UHPLC-QTOF-MS, subsequent to the implementation of digital processing. The fundamental theory underlying this approach is predicated on the observation that, although TCM materials of the same botanical origin may exhibit certain fluctuations in chemical composition due to variations in geographical origin, batch, and growth age, their overall chemical profiles remain highly similar, characterized by abundant common ion clusters; furthermore, different TCM materials possess their own specific feature ion information. Experiments confirmed that the top 100 [*t*_*R*_-*m/z*-*I*] achieve an optimal balance between identification sensitivity and specificity. Increasing the [*t*_*R*_-*m/z*-*I*] scale (e.g., to 200 or 300) did not significantly improve AR identification accuracy. Instead, it reduced matching confidence indices due to redundant information. Accordingly, this study uniformly employs the top 100 [*t*_*R*_-*m/z*-*I*] to construct digital fingerprints for AR and MR, which serve as the standard reference for subsequent identification. The digital fingerprints constructed through multibatch feature fusion and differential peak screening overcome the limitations of conventional single-marker detection methods and exhibit high robustness and specificity, providing a solid data foundation for the subsequent construction of qualitative screening and quantitative prediction models.

### 4.3 Discussion of Qualitative Screening and Quantitative Prediction Mode

Based on digital fingerprints combined with the calculation formula of IMR, this study successfully established a qualitative screening model for the adulteration of MR in AR. Meanwhile, the qualitative detection threshold was determined: when the IMR of a tested sample matched with the MR digital fingerprint is ≥10.0%, the AR sample can be judged to be adulterated with MR. Moreover, this method enables high-sensitivity qualitative screening for MR adulteration ratios as low as 5%.

Based on digital fingerprints combined with the ElasticNet algorithm, this study successfully constructed a quantitative prediction model for AR adulterated with MR. The evaluation metrics indicated that the ElasticNet model exhibited excellent performance, possessing strong predictive capability and high stability. The feature ion screening results based on RCV further revealed the chemical insights into this adulteration process. Specifically, when the |RCV| ≥ 0.02, it indicates that the feature ion has a high contribution to the quantitative prediction and can serve as a core marker for adulteration identification. For example, the response *I* values of feature ions attributed to MR (such as F101, F120, and F023) increased significantly with the elevation of the adulteration proportion; conversely, the *I* values of feature ions attributed to AR (such as F025) gradually decreased as the adulteration proportion increased. It is worth noting that an accurate quantitative evaluation of adulteration cannot rely solely on the response intensities of the aforementioned single feature ions, but must depend on the comprehensive response matrix of all 52 nonzero coefficient feature ions screened by the ElasticNet model.^38,39^

The constructed qualitative screening and quantitative prediction models were applied to the analysis of 16 batches of ABS. The results demonstrated that the quantitatively predicted MR adulteration proportions for ABS05 and ABS12 were 42.48% and 77.43%, respectively, which were highly consistent with the qualitative screening results based on IMR. Specifically, the qualitative screening results (Table 7) indicated that the IMR value of ABS05 matched against the MR digital fingerprints was 70.0%; according to the gradient patterns presented in Tables 5 and 6, when the IMR falls within the range of 60.0%–77.0%, the corresponding theoretical MR adulteration proportion is between 40% and 60%. Similarly, the IMR value of ABS12 was 84.0%, and referring to the aforementioned gradient patterns (when the IMR is between 79.0% and 85.0%), its MR adulteration proportion should be approximately 80%. Therefore, while achieving the qualitative identification of adulteration, the qualitative screening model can also preliminarily estimate the theoretical range of the MR adulteration proportion; a combined analysis with the quantitative prediction model revealed that this IMR-based estimated range was directionally consistent with the quantitatively predicted values of the ElasticNet model. Furthermore, in the quantitative prediction results, although the predicted values for blind samples ABS03, ABS06, ABS07, ABS08, ABS09, and ABS15 were greater than zero, the maximum value was merely 0.88%. Considering that the inherent quality fluctuations of AR itself may cause minor interference with the baseline of the regression model, this study further conducted a verification in combination with the qualitative screening results. The results (Table 7) demonstrated that the IMR values of the aforementioned six batches of samples matched against the MR digital fingerprints were all 0.0%. Collectively, such minor near-zero predictions were conservatively interpreted as negative.

In summary, the qualitative-quantitative coupled model constructed based on digital fingerprints in this study achieved mutual corroboration between the qualitative and quantitative results. It synergistically suggests the definite presence of MR adulteration in ABS05 and ABS12, whereas no MR adulteration was observed in the remaining samples. This strategy provides a scientific and reliable technical means for the precise identification of MR adulteration in AR.

### 4.4 Research Limitations and Future Prospects

Although this study successfully constructed digital fingerprints based on UHPLC-QTOF-MS and achieved the qualitative screening and quantitative prediction of MR adulteration in AR, the proposed method still presents several limitations that warrant further improvement. First, although the current model encompasses AR and MR samples from major producing areas, it still lacks coverage of samples from other regions and different harvesting periods; in the future, it is necessary to further expand the overall sample size to enhance the generalization capability of the digital fingerprints. Second, this study exclusively investigated the specific adulteration combination of MR incorporated into AR, and did not evaluate other easily confused adulterants. Consequently, when the IMR of a test sample is below the detection threshold and the quantitative prediction result approaches 0.0%, it merely indicates the absence of MR adulteration under the current analytical system, and does not preclude the possibility of other potential adulterants existing in the sample.

In summary, to generalize this analytical workflow from MR-specific screening to broader AR authenticity evaluation, it is necessary to establish a multiadulterant digital fingerprints library. Future research will further expand the scale of the sample database and conduct in-depth investigations into the identification methods for AR and its various potential adulterants, thereby comprehensively enhancing the robustness, reproducibility, and transferability of the entire analytical workflow.

## 5 Conclusions

In this study, a qualitative-quantitative coupled analytical model was established based on nontargeted UHPLC-QTOF-MS digital fingerprints to identify the adulteration of AR with MR. The results showed that the qualitative screening model and quantitative prediction model constructed by combining the digital fingerprints of AR and MR with the IMR screening and ElasticNet algorithm successfully screened out two MR adulterated batches, ABS05 and ABS12. Their predicted MR adulteration proportions were 42.48% and 77.43%, respectively. This study provides a scientifically reliable technical strategy for the regulatory supervision of AR adulteration with MR.

Beyond the specific detection of MR adulteration in AR, this study demonstrates that digital fingerprints derived from nontargeted mass spectrometry can serve as interpretable and quantitative chemical evidence for the authenticity evaluation of complex herbal matrices. Further expansion of reference data sets and interlaboratory validation will enable the proposed qualitative-quantitative framework to act as a transferable paradigm for data-driven quality monitoring and risk-based regulation of traditional Chinese medicines.

## Supplementary Material



## Data Availability

Data will be made available on request.

## Notes

Table S1: The detailed information on herbal materials; Table S2: The ion information on ″digital fingerprints″ of Mori Ramulus (MR); Table S3: The ion information on ″digital fingerprints″ of Astragali Radix (AR); Table S4: A total of 52 feature ions with contributions to adulteration analysis were screened based on the regression coefficient values (RCV); Table S5: Hyperparameter search spaces used for the four regression models; Table S6: Software and package versions used for regression analysis; Figure S1: ROC curve and decision threshold evaluation for screening MR adulteration (PDF)

## Abbreviations

AR: Astragali Radix
MR: Mori Ramulus
AM: *Astragalus membranaceus* (Fisch.) Bge.
AMM: *Astragalus membranaceus* (Fisch.) Bge. var. mongholicus (Bge.) Hsiao
IMR: ion mapping ratio
MS: mass spectrometry
*m*/*z*: mass-to-charge ratio
CIM: common ion matrix
SIM: specific ion matrix
TCM: Traditional Chinese Medicine
RCV: regression coefficient values
*I*: ion intensity
ABS: Astragali Radix blind samples
*t*_*R*_: retention time
