# Supplementary material for: A New Quality Control Strategy for Mori Ramulus Adulteration in Astragali Radix: A Qualitative-Quantitative Coupled Model Based on Non-Targeted Mass Spectrometry Digital Fingerprints and the ElasticNet Algorithm
Source: ACS Omega. 2026 Aug 6;11(32):48313–24. doi: 10.1021/acsomega.6c04890 (PMC13491907; doi:10.1021/acsomega.6c04890)
Supplement: Supplementary file 1 [file ao6c04890_si_001.pdf]

A New Quality Control Strategy for *Mori Ramulus* Adulteration in *Astragali Radix*: A Qualitative-Quantitative Coupled Model Based on Non-Targeted Mass Spectrometry Digital Fingerprints and the ElasticNet Algorithm

Hao Liang[1], Hui Wang[1], Xinjie Shi[1], Menghan Fan[1], Minmin Zhou[1], Bingyan Han[1], Yun Wang[1*]

1.Beijing University of Chinese Medicine, Beijing, China 100029

Table S1 The detailed information of herbal materials

Herbal materials	Batch	Sample Collection/Preparation Unit	Use	Place of origin
Astragalus membranaceus	AM01	Beijing University of Chinese Medicine	Identification verification; mixed sample	Jilin, China
Astragalus membranaceus	AM02	Beijing University of Chinese Medicine	"digital fingerprints"; mixed sample	Neimenggu, China
Astragalus membranaceus	AM03	Beijing University of Chinese Medicine	Identification verification; mixed sample	Gansu, China
Astragalus membranaceus	AM04	Beijing University of Chinese Medicine	Identification verification; mixed sample	Neimenggu, China
Astragalus membranaceus	AM05	Beijing University of Chinese Medicine	Identification verification; mixed sample	Neimenggu, China
Astragalus membranaceus	AM06	Beijing University of Chinese Medicine	"digital fingerprints"; mixed sample	Jilin, China
Astragalus membranaceus	AM07	Beijing University of Chinese Medicine	Identification verification; mixed sample	Neimenggu, China
Astragalus membranaceus	AM08	Beijing University of Chinese Medicine	"digital fingerprints"; mixed sample; model training	Gansu, China
Astragalus membranaceus	AM09	Beijing University of Chinese Medicine	Identification verification; mixed sample	Neimenggu, China
Astragalus membranaceus	AM10	Beijing University of Chinese Medicine	"digital fingerprints"; mixed sample; model training	Jilin China
Astragalus membranaceus	AM11	Beijing University of Chinese Medicine	Identification verification; mixed sample	Gansu, China
Astragalus membranaceus	AM12	Beijing University of Chinese Medicine	Identification verification; mixed sample	Jilin China
Astragalus membranaceus	AM13	Beijing University of Chinese Medicine	"digital fingerprints"; mixed sample	Gansu, China
Astragalus membranaceus	AM14	Beijing University of Chinese Medicine	Identification verification; mixed sample	Jilin, China
Astragalus membranaceus	AM15	Beijing University of Chinese Medicine	"digital fingerprints"; mixed sample	Gansu, China
Astragalus membranaceus	AM16	Beijing University of Chinese Medicine	"digital fingerprints"; mixed sample	Jilin China
Astragalus membranaceus	AM17	Beijing University of Chinese Medicine	"digital fingerprints"; mixed sample	Gansu, China
Astragalus membranaceus	AM18	Beijing University of Chinese Medicine	"digital fingerprints"; mixed sample	Neimenggu, China
Astragalus membranaceus	AM19	Beijing University of Chinese Medicine	Identification verification; mixed sample	Gansu, China
Astragalus membranaceus	AM20	Beijing University of Chinese Medicine	"digital fingerprints"; mixed sample; model training	Neimenggu, China
Astragalus membranaceus var. mongholicus	AMM01	Beijing University of Chinese Medicine	Identification verification; mixed sample	Gansu, China

Astragalus membranaceus var. mongholicus	AMM02	Beijing University of Chinese Medicine	Identification verification; mixed sample	Shanxi, China
Astragalus membranaceus var. mongholicus	AMM03	Beijing University of Chinese Medicine	"digital fingerprints"; mixed sample	Neimenggu, China
Astragalus membranaceus var. mongholicus	AMM04	Beijing University of Chinese Medicine	Identification verification; mixed sample	Shanxi, China
Astragalus membranaceus var. mongholicus	AMM05	Beijing University of Chinese Medicine	"digital fingerprints"; mixed sample	Neimenggu, China
Astragalus membranaceus var. mongholicus	AMM06	Beijing University of Chinese Medicine	Identification verification; mixed sample	Gansu, China
Astragalus membranaceus var. mongholicus	AMM07	Beijing University of Chinese Medicine	"digital fingerprints"; mixed sample	Shanxi, China
Astragalus membranaceus var. mongholicus	AMM08	Beijing University of Chinese Medicine	Identification verification; mixed sample	Neimenggu, China
Astragalus membranaceus var. mongholicus	AMM09	Beijing University of Chinese Medicine	Identification verification; mixed sample	Gansu, China
Astragalus membranaceus var. mongholicus	AMM10	Beijing University of Chinese Medicine	"digital fingerprints"; mixed sample	Shanxi, China
Astragalus membranaceus var. mongholicus	AMM11	Beijing University of Chinese Medicine	Identification verification; mixed sample; model training	Neimenggu, China
Astragalus membranaceus var. mongholicus	AMM12	Beijing University of Chinese Medicine	Identification verification; mixed sample	Shanxi, China
Astragalus membranaceus var. mongholicus	AMM13	Beijing University of Chinese Medicine	"digital fingerprints"; mixed sample	Gansu, China
Astragalus membranaceus var. mongholicus	AMM14	Beijing University of Chinese Medicine	Identification verification; mixed sample	Neimenggu, China
Astragalus membranaceus var. mongholicus	AMM15	Beijing University of Chinese Medicine	"digital fingerprints"; mixed sample	Gansu, China
Astragalus membranaceus var. mongholicus	AMM16	Beijing University of Chinese Medicine	"digital fingerprints"; mixed sample	Gansu, China
Astragalus membranaceus var. mongholicus	AMM17	Beijing University of Chinese Medicine	"digital fingerprints"; mixed sample; model training	Shanxi, China
Astragalus membranaceus var. mongholicus	AMM18	Beijing University of Chinese Medicine	Identification verification; mixed sample	Gansu, China
Astragalus membranaceus var. mongholicus	AMM19	Beijing University of Chinese Medicine	"digital fingerprints"; mixed sample	Neimenggu, China
Astragalus membranaceus var. mongholicus	AMM20	Beijing University of Chinese Medicine	"digital fingerprints"; mixed sample; model training	Neimenggu, China
Mori Ramulus	MR01	Beijing University of Chinese Medicine	Identification verification; mixed sample	Sichuan, China
Mori Ramulus	MR02	Beijing University of Chinese Medicine	"digital fingerprints"; mixed sample	Jiangsu, China
Mori Ramulus	MR03	Beijing University of Chinese Medicine	Identification verification; mixed sample	Zhejiang, China
Mori Ramulus	MR04	Beijing University of Chinese Medicine	Identification verification; mixed sample; model training	Jiangsu, China
Mori Ramulus	MR05	Beijing University of Chinese Medicine	"digital fingerprints"; mixed sample; model training	Jiangsu, China
Mori Ramulus	MR06	Beijing University of Chinese Medicine	Identification verification; mixed sample; model training	Sichuan, China
Mori Ramulus	MR07	Beijing University of Chinese Medicine	Identification verification; mixed sample	Jiangsu, China
Mori Ramulus	MR08	Beijing University of Chinese Medicine	"digital fingerprints"; mixed sample;	Zhejiang, China

Mori Ramulus	MR09	Beijing University of Chinese Medicine	Identification verification; mixed sample	Zhejiang, China
Mori Ramulus	MR10	Beijing University of Chinese Medicine	"digital fingerprints"; mixed sample;	Sichuan, China
Mori Ramulus	MR11	Beijing University of Chinese Medicine	Identification verification; mixed sample	Zhejiang, China
Mori Ramulus	MR12	Beijing University of Chinese Medicine	Identification verification; mixed sample	Sichuan, China
Mori Ramulus	MR13	Beijing University of Chinese Medicine	"digital fingerprints"; mixed sample	Sichuan, China
Mori Ramulus	MR14	Beijing University of Chinese Medicine	Identification verification; mixed sample	Jiangsu, China
Mori Ramulus	MR15	Beijing University of Chinese Medicine	"digital fingerprints"; mixed sample	Zhejiang, China
Mori Ramulus	MR16	Beijing University of Chinese Medicine	Identification verification; mixed sample	Sichuan, China
Mori Ramulus	MR17	Beijing University of Chinese Medicine	"digital fingerprints"; mixed sample	Jiangsu, China
Mori Ramulus	MR18	Beijing University of Chinese Medicine	Identification verification; mixed sample	Sichuan, China
Mori Ramulus	MR19	Beijing University of Chinese Medicine	Identification verification; mixed sample	Zhejiang, China
Mori Ramulus	MR20	Beijing University of Chinese Medicine	Identification verification; mixed sample	Sichuan, China
Mori Ramulus	MR21	Beijing University of Chinese Medicine	"digital fingerprints"; mixed sample	Zhejiang, China
Mori Ramulus	MR22	Beijing University of Chinese Medicine	Identification verification; mixed sample;	Jiangsu, China
Mori Ramulus	MR23	Beijing University of Chinese Medicine	Identification verification; mixed sample	Zhejiang, China
Mori Ramulus	MR24	Beijing University of Chinese Medicine	"digital fingerprints"; mixed sample	Jiangsu, China
Mori Ramulus	MR25	Beijing University of Chinese Medicine	"digital fingerprints"; mixed sample	Sichuan, China
0 % MR	MixMR01-1	self-preparation	Identification verification	—
0 % MR	MixMR01-2	self-preparation	Identification verification	—
0 % MR	MixMR01-3	self-preparation	Identification verification	—
5 % MR	MixMR02-1	self-preparation	Identification verification; model training	—
5 % MR	MixMR02-2	self-preparation	Identification verification; model training	—
5 % MR	MixMR02-3	self-preparation	Identification verification; model training	—
10 % MR	MixMR03-1	self-preparation	Identification verification; model training	—
10 % MR	MixMR03-2	self-preparation	Identification verification; model training	—
10 % MR	MixMR03-3	self-preparation	Identification verification; model training	—
20 % MR	MixMR04-1	self-preparation	Identification verification; model training	—
20 % MR	MixMR04-2	self-preparation	Identification verification; model training	—
20 % MR	MixMR04-3	self-preparation	Identification verification; model training	—
40 % MR	MixMR05-1	self-preparation	Identification verification; model training	—
40 % MR	MixMR05-2	self-preparation	Identification verification; model training	—
40 % MR	MixMR05-3	self-preparation	Identification verification; model training	—

60 % MR	MixMR06-1	self-preparation	Identification verification; model training	—
60 % MR	MixMR06-2	self-preparation	Identification verification; model training	—
60 % MR	MixMR06-3	self-preparation	Identification verification; model training	—
80 % MR	MixMR07-1	self-preparation	Identification verification; model training	—
80 % MR	MixMR07-2	self-preparation	Identification verification; model training	—
80 % MR	MixMR07-3	self-preparation	Identification verification; model training	—
100 % MR	MixMR08-1	self-preparation	Identification verification	—
100 % MR	MixMR08-2	self-preparation	Identification verification	—
100 % MR	MixMR08-3	self-preparation	Identification verification	—
Blind market AR materials	ABS01	Company Code: KMYY	Identification analysis	—
Blind market AR materials	ABS02	Company Code:AGME	Identification analysis	—
Blind market AR materials	ABS03	Company Code: FDH	Identification analysis	—
Blind market AR materials	ABS04	Company Code:BZJC	Identification analysis	—
Blind market AR materials	ABS05	Company Code: YNBY	Identification analysis	—
Blind market AR materials	ABS06	Company Code: LYS	Identification analysis	—
Blind market AR materials	ABS07	Company Code: MNR	Identification analysis	—
Blind market AR materials	ABS08	Company Code: QCT	Identification analysis	—
Blind market AR materials	ABS09	Company Code: BX	Identification analysis	—
Blind market AR materials	ABS10	Company Code: QLX	Identification analysis	—
Blind market AR materials	ABS11	Company Code: LST	Identification analysis	—
Blind market AR materials	ABS12	Company Code:AGBCJ	Identification analysis	—
Blind market AR materials	ABS13	Company Code: JHT	Identification analysis	—
Blind market AR materials	ABS14	Company Code: PZD	Identification analysis	—
Blind market AR materials	ABS15	Company Code: ZCT	Identification analysis	—
Blind market AR materials	ABS16	Company Code: BCY	Identification analysis	—

Table S2 The ion information of "digital fingerprints" of *Mori Ramulus*

t_R	*m/z*	*I*	t_R	*m/z*	*I*	t_R	*m/z*	*I*
25.29	270.32	4016528	23.04	323.09	45038	15.91	341.14	26373
24.74	423.18	301156	39.10	642.57	44474	26.94	608.40	25161

12.47	595.35	262368	23.80	711.25	41786	22.54	1354.49	25120
29.36	679.26	212218	28.72	423.18	41784	22.53	973.35	25066
39.07	686.59	198333	22.53	686.26	41773	22.54	1137.40	24621
22.50	487.18	187194	11.50	501.12	41258	32.36	467.39	24128
23.60	693.23	140095	22.63	353.10	40510	22.52	147.04	24011
19.33	547.20	125985	32.73	467.39	39123	10.92	330.26	23988
19.61	549.21	108688	21.18	367.12	37798	23.22	517.19	23221
27.97	365.10	101163	23.13	721.23	37223	22.55	455.15	23130
17.88	531.20	85273	23.06	355.12	36654	19.33	385.13	22895
29.47	421.16	81989	8.12	492.32	35590	40.40	421.35	22865
20.80	367.12	81223	26.13	453.19	35556	41.77	1054.30	22716
22.55	323.13	74369	16.87	200.38	35417	30.36	663.26	22657
39.73	700.57	69148	25.78	693.23	34987	15.46	565.21	21180
26.22	761.30	68793	37.02	425.38	34856	19.62	713.26	20907
9.00	492.32	68704	41.71	481.37	33056	17.86	713.26	20472
25.56	693.23	67143	38.98	437.34	32743	24.17	271.32	20446
39.61	568.43	64316	20.81	369.13	32479	17.89	549.21	19989
22.55	691.22	64168	23.64	341.14	31510	32.23	400.38	19926
23.06	517.19	63908	18.09	531.20	31349	15.25	826.31	19666
19.53	547.20	59959	4.36	245.08	31217	14.72	547.20	18731
39.66	499.38	59953	9.16	461.14	31201	19.32	711.24	18444
25.76	695.25	59240	37.77	1432.12	30951	38.78	642.57	17907
33.11	439.36	58259	25.29	270.61	30261	41.30	483.38	17300
41.85	416.37	54909	23.38	353.10	30120	23.17	439.18	17267
8.51	492.32	53333	22.91	487.18	29116	37.41	593.28	16993
19.88	549.21	52661	34.49	457.37	28801	23.06	681.23	16892
25.59	323.13	51106	41.29	423.36	27953	24.73	311.06	16867
41.86	433.37	50339	25.31	695.25	27849	17.96	547.20	16832
18.96	351.12	49153	35.57	625.27	27700	23.43	751.24	16416
36.07	609.27	47242	27.27	298.35	27402	18.81	517.19	16030
25.62	487.18	47074	35.14	609.27	26486	31.64	421.16	16012
38.96	497.36	45600	—	—	—	—	—	—

Table S3 The ion information of "digital fingerprints" of *Astragali Radix*

t_R	*m/z*	*I*	t_R	*m/z*	*I*	t_R	*m/z*	*I*
41.64	778.54	149136	35.84	693.37	33034	36.83	786.60	18046
8.26	447.13	145608	19.89	437.34	32811	9.13	217.15	17832
37.84	804.56	142314	37.96	796.55	32157	19.31	1031.55	17801
41.82	778.54	137756	34.41	808.59	31756	36.25	510.43	17781
12.88	285.08	116615	19.89	629.41	31731	23.78	671.42	16978
10.35	533.13	115503	34.44	808.57	29983	39.11	551.50	16797
39.77	798.57	108871	22.78	851.48	29910	18.37	437.34	16691
17.04	269.08	107960	38.76	778.54	28873	22.50	941.52	16314
40.65	796.56	104012	41.68	954.62	27429	9.71	533.13	16212
38.21	796.55	99786	18.62	785.47	27240	23.79	869.49	16095
22.76	869.49	98278	40.84	555.44	26531	34.16	671.46	15888
39.85	772.56	86150	38.70	958.65	23725	36.52	742.42	15724
40.39	772.59	81388	35.30	545.43	23218	8.59	477.14	15694
20.65	943.53	77995	38.29	804.56	22869	8.26	893.25	15416
13.65	517.13	77852	17.54	299.09	22727	37.34	786.60	14976
11.80	431.13	75882	34.86	545.43	22510	34.04	309.24	14774
22.77	671.42	73386	37.50	814.56	22105	35.33	510.43	14598
26.33	518.32	68933	10.50	563.14	21690	13.41	315.09	14282
40.48	796.56	66535	38.23	794.54	21214	19.90	1240.71	14138
12.79	301.11	63141	17.57	947.52	21189	40.31	770.54	14085
41.51	798.57	58146	35.38	812.55	20699	33.56	573.49	13944
8.26	285.08	53887	14.25	549.16	20603	38.79	804.56	13732
39.86	796.55	52855	15.66	947.53	20544	27.13	713.43	13636
22.76	1303.73	52113	40.81	830.68	20502	9.66	301.07	13618
22.75	437.34	44871	18.61	437.34	20088	40.10	780.56	13609
6.08	285.19	43644	39.17	790.57	19955	38.71	794.54	13563
38.45	958.65	42534	35.90	833.52	19778	11.66	489.14	13443
8.48	289.18	42329	38.23	818.54	19619	10.35	285.08	13428
28.02	478.29	40920	12.79	463.16	19515	38.25	613.45	13417
19.90	827.48	40790	38.96	778.54	19112	19.90	1479.89	13355
27.53	520.34	39556	37.86	818.54	18855	40.24	808.59	13296

17.53	301.11	36918	38.25	958.66	18781	18.38	785.47	13241
11.82	269.08	36262	38.20	778.54	18275	41.76	615.48	13049
38.81	529.44	35372	—	—	—	—	—	—

Table S4 A total of 52 feature ions with contributions to adulteration analysis were screened based on the regression coefficient values (RCV)

Feature ion	t_R	*m/z*	RCV	Ion assignment	Feature Ion	t_R	*m/z*	RCV	Ion assignment
F001	4.36	245.0803	0.0114	MR	F095	23.64	341.1387	0.0114	MR
F008	8.51	492.3157	0.0148	MR	F098	23.80	711.2459	0.0063	MR
F010	9.00	492.3170	0.0023	MR	F100	24.73	311.0550	0.0035	MR
F011	9.13	217.1547	-0.0018	AR	F101	24.74	423.1806	0.0269	MR
F018	10.92	330.2637	0.0066	MR	F102	25.29	270.3163	0.0021	MR
F023	12.47	595.3496	0.0218	MR	F104	25.31	695.2493	0.0043	MR
F025	12.79	463.1598	-0.0180	AR	F105	25.56	693.2346	0.0107	MR
F030	14.72	547.1961	0.0005	MR	F106	25.59	323.1279	0.0215	MR
F042	17.89	549.2110	0.0024	MR	F107	25.62	487.1757	0.0102	MR
F047	18.61	437.3410	-0.0015	AR	F108	25.76	695.2482	0.0059	MR
F050	18.96	351.1224	0.0063	MR	F109	25.78	693.2334	0.002	MR
F054	19.33	547.1973	0.0029	MR	F110	26.13	453.1911	0.0024	MR
F055	19.53	547.1976	0.0026	MR	F111	26.22	761.2971	0.0013	MR
F056	19.61	549.2122	0.0094	MR	F116	27.53	520.3400	-0.0053	AR
F058	19.88	549.2130	0.0006	MR	F117	27.97	365.1016	0.0151	MR
F060	19.89	629.4057	-0.0083	AR	F120	29.36	679.2556	0.0243	MR
F064	20.65	943.5319	-0.0211	AR	F121	29.47	421.1647	0.0017	MR
F068	22.50	941.5151	-0.0093	AR	F125	32.36	467.3875	-0.0005	AR
F072	22.53	686.2610	0.0060	MR	F126	32.73	467.3876	0.0011	MR
F076	22.55	455.1482	0.0053	MR	F141	35.90	833.5231	-0.0002	AR
F077	22.55	323.1271	0.0055	MR	F142	36.07	609.2715	0.0058	MR
F078	22.63	353.1017	0.0059	MR	F158	38.25	958.6551	0.0008	MR
F083	22.78	851.4847	-0.0112	AR	F166	38.79	804.5550	-0.0011	AR
F088	23.06	517.1865	0.0062	MR	F177	39.73	700.5748	0.0009	MR
F090	23.17	439.1753	0.0004	MR	F191	41.30	483.3834	0.0017	MR
F094	23.60	693.2348	0.0204	MR	F197	41.76	615.4800	-0.0015	AR

Table S5 Hyperparameter search spaces used for the four regression models

Model	Hyperparameter	Search space
OPLS-R	opls__n_components	1, 2, and 3
	pls__n_components	1, 2, 3, and 4
SVR	svr__kernel	linear
	svr__C	0.1, 1, 10, and 100
RFR	rf__n_estimators	50, 100, and 200
	rf__max_depth	None, 10, and 20
Elastic Net	l1_ratio	0.01, 0.10, 0.30, 0.50, 0.70, 0.90, 0.95, 0.99, and 1.00
	alpha	100 logarithmically spaced values for each l1_ratio, ranging from alpha_max to 0.001 alpha_max; values were data- and fold-dependent

Table S6 Software and package versions used for regression analysis

Software or package	Version
Python	3.12.7
conda	24.9.2
pandas	2.2.2
NumPy	1.26.4
scikit-learn	1.5.1
SciPy	1.13.1
joblib	1.4.2
threadpoolctl	3.5.0

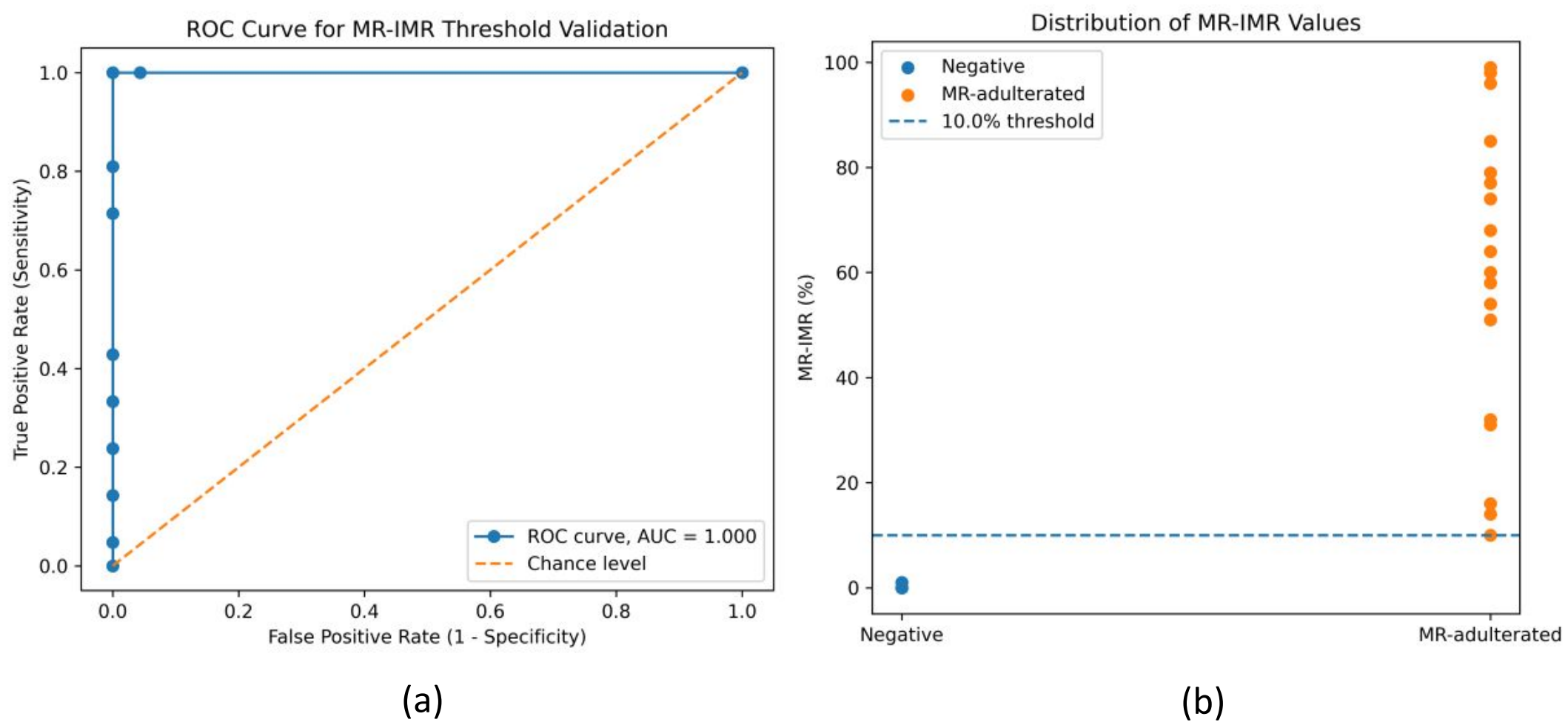

Figure S1 ROC curve and decision threshold evaluation for screening MR adulteration
(a) ROC curve analysis of MR-IMR for distinguishing AR from MR adulterated samples; (b) decision threshold (MR-IMR $\geqslant$ 10.0%) evaluation for screening MR adulteration
